# High prevalence of increased sitosterol levels in hypercholesterolemic children suggest underestimation of sitosterolemia incidence

**DOI:** 10.1371/journal.pone.0238079

**Published:** 2020-08-26

**Authors:** Joon Hee Lee, Da Young Song, Sun-Hee Jun, Sang Hoon Song, Choong Ho Shin, Chang-Seok Ki, Kyunghoon Lee, Junghan Song

**Affiliations:** 1 Department of Laboratory Medicine, Seoul National University College of Medicine, Seoul, Korea; 2 Department of Laboratory Medicine, Seoul National University Bundang Hospital, Seongnam, Korea; 3 Department of Laboratory Medicine, Seoul National University Hospital, Seoul, Korea; 4 Department of Pediatrics, Seoul National University Children's Hospital, Seoul, Korea; 5 GC Genome, Yongin, Korea; Università degli Studi di Milano, ITALY

## Abstract

**Background:**

Sitosterolemia is an inherited lipid disorder which presents with elevated serum sitosterol and can result in an increased risk of premature cardiovascular disease. However, sitosterol cannot be accurately measured by routine diagnostic assays, meaning that sitosterolemia diagnosis can often be difficult, especially with many clinical features overlapping with familial hypercholesterolemia. With such complications resulting in increasing reports of misdiagnosis, the prevalence of sitosterolemia is predicted to be much higher than previously reported.

**Methods:**

Gas chromatography-mass spectrometry was utilized to measure sitosterol levels of normocholesterolemic and hypercholesterolemic children. Subsequently, an epidemiologically determined cutoff level of sitosterol was calculated and applied to estimate the prevalence of children with increased sitosterol and identify potential sitosterolemia patients. Massively parallel sequencing was used to confirm the diagnosis in suspected patients.

**Results:**

Samples from 109 normocholesterolemic and 220 hypercholesterolemic were tested for phytosterols. Sitosterol and campesterol levels were significantly increased in hypercholesterolemic children (mean 22.0±45.9 μmol/L for sitosterol and 26.0±32.8 μmol/L for campesterol) compared to normocholesterolemic children (mean 12.1±4.9 μmol/L for sistosterol and 14.8±6.7 μmol/L for campesterol). Via application of a cutoff of 35.9 μmol/L, the prevalence rates for increased and overtly increased sitosterol in hypercholesterolemic children were 6.4% and 1.4% respectively. Furthermore, 3 suspected sitosterolemia patients were identified, with 2 patients receiving molecular confirmation for sitosterolemia diagnosis.

**Conclusions:**

Our findings reaffirm that the prevalence of sitosterolemia is probably much higher than previously reported, which also indicates the significant risk of misdiagnosis of sitosterolemia with familial hypercholesterolemia. Special lipid testing including sitosterol, especially in children with uncontrolled hypercholesterolemia, is recommended in children in order to identify potential sitosterolemia patients that would otherwise be neglected.

## Introduction

Sitosterolemia is a rare, autosomal recessive lipid disorder with heterogeneous clinical presentations, ranging from asymptomatic to premature cardiovascular disease, xanthomatosis, and/or hematologic manifestations [1]. It is characterized by an increase in sitosterol, which is a phytosterol, or plant sterol, similar to cholesterol but differing in carbon side chain composition. Due to this structural analogy, it is difficult to measure phytosterol levels with standard diagnostic assays. For example, enzymatic colorimetry, which reacts to the C-5 double bond or presence of 3β-hydroxyl group within the sterol architecture, cannot differentiate between cholesterol and other phytosterols because the reactive groups are present on both types of sterols [2, 3]. Thus, specialized techniques such as mass spectrometry (MS) are required to properly measure phytosterols.

The pathogenesis of sitosterolemia is defined by pathogenic variants in either the *ABCG5* and/or *ABCG8* genes [4], which results in increased intestinal absorption and decreased biliary extraction of plant sterols. Normally, ingested cholesterol in the intestinal lumen is absorbed by the Nieman Pick C1 Like 1 (NPC1L1) transporter [5] of the enterocyte, then esterified by the acetyl-sterol O-acyltransferase 2 (SOAT2) [6]. Esterified cholesterol is transported to the liver in the form of chylomicrons, while unesterified cholesterol is secreted back into the lumen via ABCG5/ABCG8 efflux transporters [7]. Sitosterol shares the aforementioned influx/efflux transporters with cholesterol, but these enzymes have a much higher affinity for cholesterol, which results in significant sitosterol excretion with minimal absorption. In the pathogenic circumstances of sitosterolemia, variants in the *ABCG5* or *ABCG8* genes lead to reduced sitosterol excretion [4], consequently resulting in increased absorption of sitosterol.

Although an absolute consensus has yet to be reached, the prevalence of sitosterolemia has been reported to be as low as <1/1,000,000~5,000,000 [8, 9]. This perceived low prevalence, along with heterogeneous clinical presentations overlapping with familial hypercholesterolemia, in conjunction with the inability of conventional cholesterol assays to discriminate phytosterols from cholesterol all contribute to the difficulty of accurately diagnosing sitosterolemia. Ironically, there is an effective treatment in the form of ezetimibe, which inhibits sitosterol absorption in the intestines, and has been proven to both lower sitosterol levels and alleviate symptoms [1, 2, 10]. Current pediatric guidelines recommend primary screening at 9–11 years followed by secondary tests at 17–21 years of age [11], but these guidelines do not encompass phytosterol testing with great depth, consequently resulting in an increased risk of missing the rarer, inherited disorders. However, with increasing reports of sitosterolemia being misdiagnosed as components of familial hypercholesterolemia [9, 12], questions are consistently being raised about the true prevalence and possible underdetection of sitosterolemia.

In the current report, we investigate the association between cholesterol and sitosterol levels in a Korean pediatric population, and via application of an epidemiologically determined cutoff, demonstrate an approximate prevalence of children with increased sitosterol and also potential sitosterolemia patients. In addition, massively parallel sequencing is utilized to confirm sitosterolemia diagnosis in two hypercholesterolemic patients with increased sitosterol.

## Materials and methods

### Patients

Pediatric samples were collected from Seoul National University Children’s Hospital and Seoul National University Bundang Hospital between April 2018 and October 2018. The study was approved by the institutional review board of Seoul National University Hospital. Due to the retrospective nature of the study and analysis utilizing anonymous data from residual clinical samples, informed consent was waived for subjects for which only phytosterols were measured. For hypersitosterolemic subjects recommended for additional molecular testing, clinical data disclosure was approved after informed, written consent was obtained from the parents/legal guardians of all applicable children.

Normocholesterolemic (<5.18 mmol/L [= 200 mg/dL]) pediatric samples were obtained from children (≤18 years of age) undergoing health examinations or routine vaccinations. Hypercholesterolemic (>6.48 mmol/L [= 250 mg/dL]) pediatric samples were obtained from children from both inpatient wards and outpatient clinics. Exclusion criteria included patients diagnosed with either malignant neoplasms or severe congenital disorders, based on the rationale that increased sitosterol would not alter their treatment.

### Determination of abnormal sitosterol cutoff value

Sitosterol values from the normocholesterolemic group were used to establish a cutoff value for use as an arbitrary cutoff to distinguish between normal control and sitosterolemic patient. In this study, the upper cutoff value for sitosterol was defined as the (median + 5SD) value of the normocholesterolemic group.

### Measurement of plant sterols using gas chromatography-mass spectrometry

Serum sitosterol and campesterol, another plant sterol commonly elevated in sitosterolemia, were measured using GC-MS. A detailed description of the sample preparation procedure, GC-MS instrument and conditions, and assay performance have been published in a previous study [13]. In brief, samples were prepared by a sequential process consisting of proteolysis, liquid-liquid extraction, and derivatization. A HP 6890N gas chromatograph, HP 5975 mass spectrometer, and a HP-5MS capillary column (all from Agilent Technologies, Santa Clara, CA, USA) were used for GC-MS analysis.

### Massively parallel and Sanger sequencing

Following informed consent, massively parallel sequencing was performed on patients with abnormally high sitosterol levels. The molecular panel consisted of 31 target genes (Table 1) associated with familial dyslipidemia. Target enrichment was achieved via hybridization with oligonucleotide probes, and sequencing was conducted on an Illumina MiSeqDX (Illumina, San Diego, CA, USA) platform. Sequencing reads were aligned to the reference human genome (hg19). The bioinformatic pipeline consisted of alignment with BWA and variant calling via GATK. Obtained variants were compared with ClinVar database, Human Gene Mutation Database (HGMD), Korean Reference Genome Database (KRGDB), and Genome Aggregation Database (gnomAD). MutationTaster, SIFT, and Polyphen2 were used for in-silico prediction (ISP) of obtained variants.

**Table 1:** List of genes included in the familial dyslipidemia panel.

Gene	Disease	Inheritance	Gene	Disease	Inheritance
*ABCA1*	Tangier disease; Familial hypoalphalipoproteinemia; High density lipoprotein deficiency	AR; AD	*GPD1*	Transient infantile hypertriglyceridemia	AR
*ABCG5*	Sitosterolemia	AR	*GPIHBP1*	Hyperlipoproteinemia type 1D	AR
*ABCG8*	Sitosterolemia	AR	*LCAT*	Familial *LCAT* deficiency	AR
*ALMS1*	Alstrom syndrome	AR	*LDLR*	Familial hypercholesterolemia	AD
*ANGPTL3*	Familial hypobetalipoproteinemia; Familial combined hypolipidemia	AR	*LDLRAP1*	Familial hypercholesterolemia	AR
*APOA1*	Hypoalphalipoproteinemia	AD	*LIPA*	Cholesteryl ester storage disease; Wolman disease	AR
*APOA2*	Apolipoprotein A-II deficiency	AD	*LIPC*	Hepatic lipase deficiency	AR
*APOA5*	Late-onset hyperchylomicronemia; Hypertriglyceridemia	AD	*LMF1*	Combined lipase deficiency	AR
*APOB*	Familial hypercholesterolemia; Hypobetalipoproteinemia	AD; AR	*LPL*	Lipoprotein lipase deficiency; familial combined hyperlipidemia	AR; AD
*APOC2*	Hyperlipoproteinemia type Ib; Apolipoprotein C-II deficiency	AR	*MTTP*	Abetalipoproteinemia	AR
*APOC3*	Apolipoprotein C-III deficiency	AD	*PCSK9*	Familial hypercholesterolemia	AD
*APOE*	Hyperlipoproteinemia type III	AD	*SAR1B*	Chylomicron retention disease	AR
*CETP*	Hyperalphalipoproteinemia	AD	*SCARB1*	*SCARB1* deficiency (with elevated HDL levels)	AD/AR
*CREB3L3*	Hypertriglyceridaemia	AD	*SLCO1B1*	Response to statin therapy	
*CYP7A1*	Hyperlipidemia	AR	*STAP1*	Familial hypercholesteremia	AD
*CYP27A1*	Cerebrotendinous xanthomatosis	AR			

Abbreviations: AR, autosomal recessive; AD, autosomal dominant.

Sanger sequencing was conducted on patient samples for confirmation of suspected variants, and on family members for familial association analyses.

### Statistical analysis

All statistical analyses were done using MedCalc version 14.8.1 (MedCalc Software, Mariakerke, Belgium), and statistical significance was defined as *P*<0.05. For comparison between the normocholesterolemic and hypercholesterolemic groups, a Chi-squared test was used to compared categorical data and a Mann-Whitney U test was used to compare numerical data, including sitosterol and campesterol levels.

## Results

### Study population

Samples from 109 normocholesterolemic and 220 hypercholesterolemic children were collected. Basic characteristics of the study groups are shown in Table 2. The normocholesterolemic group (mean age 6.8 years) was slightly younger than the hypercholesterolemic group (mean age 9.8 years), but there was no significant difference in sex distribution between the two study groups.

**Table 2:** Basic characteristics of study groups.

	Normocholesterolemic group (N = 109)	Hypercholesterolemic group (N = 220)	p-value
Sex			0.833
Male	65 (59.6%)	127 (57.7%)	
Female	44 (40.4%)	93 (42.3%)	
Age (years)	6.8 ± 4.4	9.8 ± 4.9	<0.001
Cholesterol (mmol/L)	4.03 ± 0.56	8.39 ± 2.60	<0.001

### Higher sitosterol and campesterol levels in hypercholesterolemic patients and determination of abnormal sitosterol cutoff value

**Fig 1:**
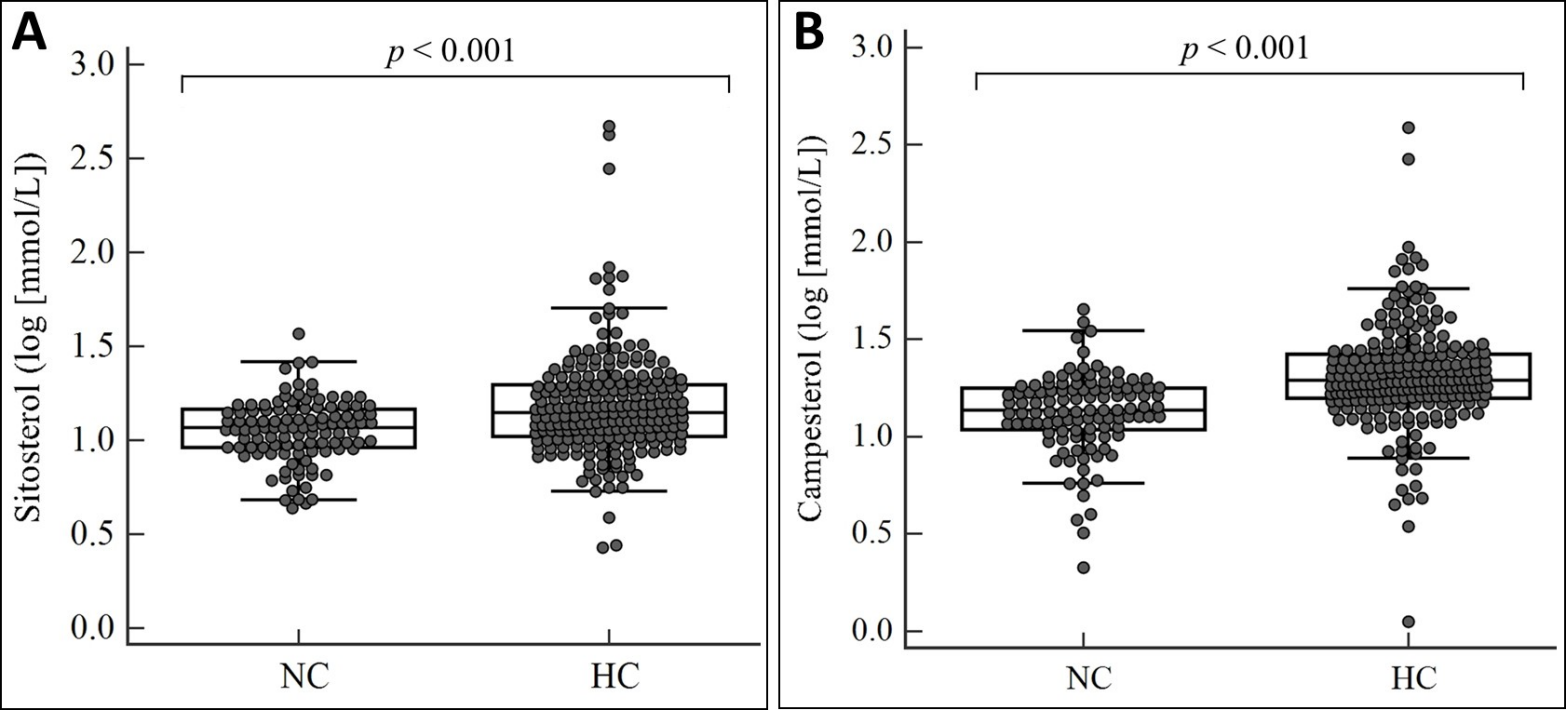
Comparison of sitosterol (A) and campesterol (B) levels between normocholesterolemic (NC) and hypercholesterolemic (HC) children.

Sitosterol levels were significantly increased in the hypercholesterolemic group (mean 22.0±45.9 μmol/L) compared to the normocholesterolemic group (mean 12.1±4.9 μmol/L) (Fig 1A), showing a higher proportion of patients from the hypercholesterolemic group with increased sitosterol levels (*P* < 0.001). From the sitosterol distribution of the normocholesterolemic group, an arbitrary value (median + 5SD) of 35.9 μmol/L was established as the abnormal cutoff for sitosterol. A total of 15 patients (14 hypercholesterolemic patients and 1 normocholesterolemic patient) had sitosterol levels above the cutoff (>35.9 μmol/L), and the percentage of patients above the sitosterol cutoff were higher in the hypercholesterolemic group (6.4%) than the normocholesterolemic group (0.9%). The distribution of campesterol was similar to that of sitosterol (Fig 1B), with significantly higher concentrations observed in the hypercholesterolemic group (mean 26.0±32.8 μmol/L) compared to the normocholesterolemic group (mean 14.8±6.7 μmol/L) (*P* < 0.001).

### Hypersitosterolemic patients

Basic clinical data of the 15 patients with sitosterol levels above the cutoff are shown in Table 3. Three patients (HC1, HC2, and HC3) showed overtly increased sitosterol levels, and additional laboratory data and clinical history of these patients are provided in Table 4.

**Table 3:** Basic clinical data of hypersitosterolemic patients (in decreasing order of sitosterol concentrations).

Patient	Sex	Age (yr)	Cholesterol (mmol/L)	Sitosterol (μmol/L)	Campesterol (μmol/L)	Clinical diagnosis
HC1	F	18	8.81	468.1	388.9	NS
HC2	F	7	7.46	424.4	267.1	Hypercholesterolemia (on statin therapy)
HC3	F	0.5	8.21	278.3	81.7	BPD, R/O TPN-associated cholestasis
HC4	F	11	6.89	82.9	44.4	Encephalomyelitis
HC5	M	11	11.76	74.6	76.9	ESRD
HC6	F	10	9.40	73.5	53.3	Alagille syndrome
HC7	M	7	6.63	73.0	83.3	Hypercholesterolemia
HC8	M	16	24.76	63.3	94.2	NS
HC9	F	18	10.75	50.5	71.1	Alagille syndrome
HC10	F	13	10.52	47.6	73.2	LFT abnormality
HC11	F	13	7.46	46.9	27.7	Juvenile dermatomyositis
HC12	F	6	16.37	44.9	58.9	NS
HC13	M	8	13.16	37.1	57.6	NS
HC14	M	3	6.63	37.0	43.8	Encephalitis
NC1	M	1	4.48	36.8	45.2	Concealed genitalia

Abbreviations: HC, hypercholesterolemic group; NC, normocholesterolemic group; NS, nephrotic syndrome; BPD, bronchopulmonary dysplasia; TPN, total parenteral nutrition; ESRD, end-stage renal disease; LFT, liver function test

**Table 4:** Additional data of three overtly hypersitosterolemic patients.

Patient	Sex	Age (yr)	Clinical diagnosis	Sitosterol (μmol/L)	Hb (mmol/L)	WBC (x10^9^/L)	PLT (x10^9^/L)	LDL-C (mmol/L)	HDL-C (mmol/L)	TG (mmol/L)	Presence of xanthomas	Associated family history
HC1	F	18	NS	468.1	8.5	5.48	256	6.24	1.52	1.75	No	No
HC2	F	7	Hypercholesterolemia (on statin therapy)	424.4	6.8	6.03	304	5.41	1.17	0.77	No	Yes
HC3	F	0.5	BPD, R/O TPN-associated cholestasis	278.3	8.3	13.53	121	6.55	NT	2.41	No	No

Abbreviations: Hb, hemoglobin; WBC, white blood cell count; PLT, platelet; LDL-C, low-density lipoprotein cholesterol; HDL-C, high-density lipoprotein cholesterol; TG, triglyceride; NT, not tested; NS, nephrotic syndrome; BPD, bronchopulmonary dysplasia; TPN, total parenteral nutrition

### Sitosterolemia diagnosis confirmed by genomic analysis in 2 patients

Informed consent was obtained from 2 (HC1, HC2) of the 3 overtly hypersitosterolemic patients. Massively parallel sequencing results showed variants in the *ABCG5* gene (NM_022436.2) for HC1 and *ABCG8* gene (NM_022437.2) in HC2, both of which are known causative genes for sitosterolemia.

**Fig 2:**
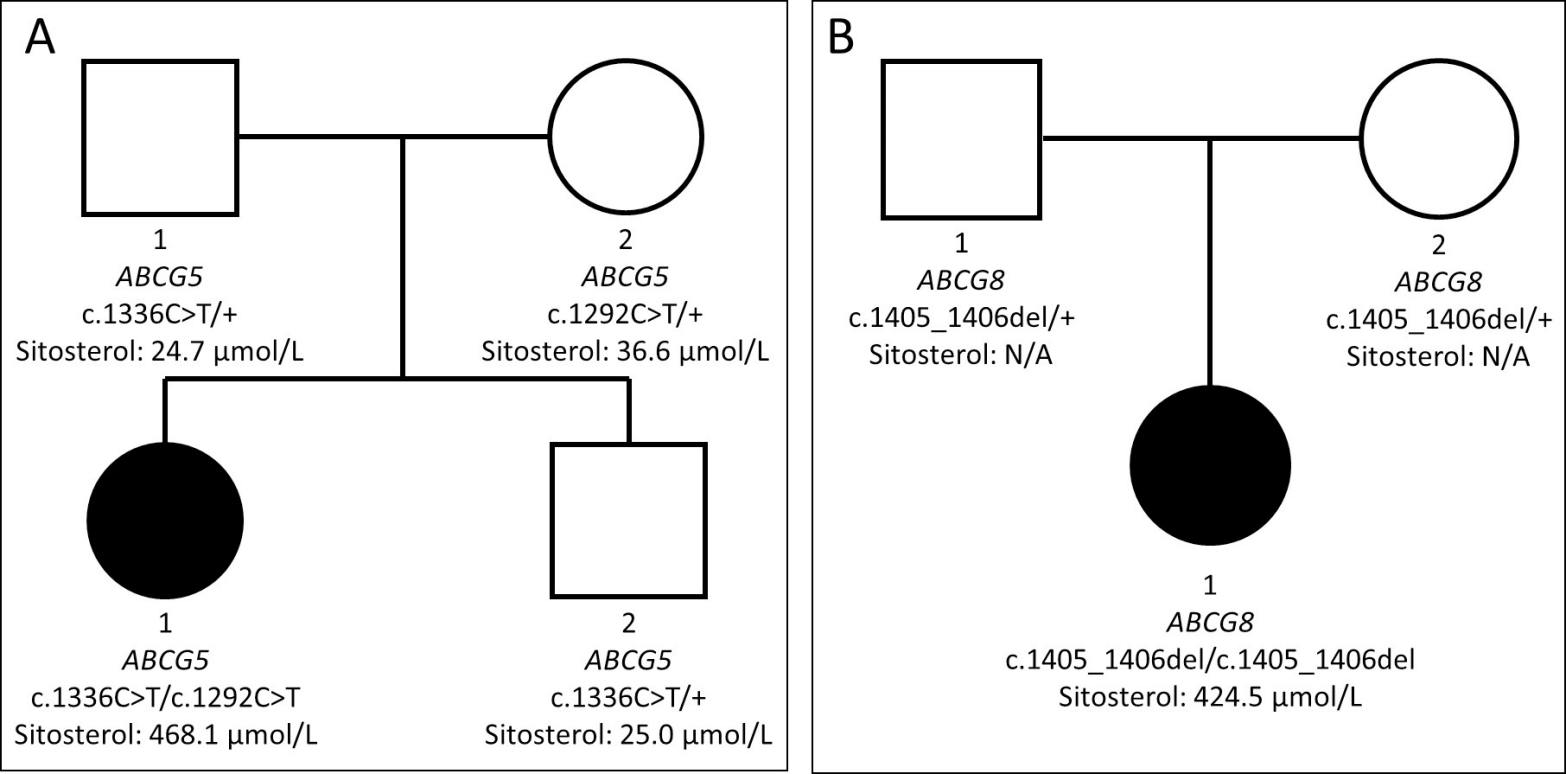
Family trees depicting variants in the ABCG5 gene of patient HC1 (A) and in the ABCG8 gene of patient HC2 (B).

Patient HC1 (F/18) was heterozygous with two different *ABCG5* variants, c.1292C>T (p.Pro431Leu) and c.1336C>T (p.Arg446*), which were inherited from her mother and father respectively (Fig 2A and Table 5). According to the American College of Medical Genetics and Genomics/Association for Molecular Pathology (ACMG/AMP) standards and guidelines for the interpretation of sequence variants, the variants are classified as a variant of uncertain significance (VUS) and likely pathogenic (LP) variant respectively. Sitosterol results of other family members (parents and younger brother) showed no overtly increased sitosterol levels, although her mother had sitosterol levels (36.6 μmol/L) mildly above the predetermined sitosterol cutoff value (35.9 μmol/L).

**Table 5:** Pathogenic variants in two overtly hypersitosterolemic patients.

Patient	Gene	Exon	Nucleotide (NCBI RefSeq)	Amino acid change	Parental origin	Classification of pathogenicity^a^
HC1	ABCG5	9	c.1292C>T (NM_022436.2)	p.Pro431Leu	Maternal	VUS
	ABCG5	10	c.1336C>T (NM_022436.2)	p.Arg446*	Paternal	LP
HC2	ABCG8	9	c.1405_1406del (NM_022437.2)	p.Ser469Glnfs*12	Paternal & maternal	LP

Abbreviations: VUS, variant of uncertain significance; LP, likely pathogenic

^a^ Based on American College of Medical Genetics and Genomics/Association for Molecular Pathology (ACMG/AMP) standards and guidelines for the interpretation of sequence variants

Patient HC2 (F/8) was homozygous for the *ABCG8* variant c.1405_1406del (p.Ser469Glnfs*12), with her parents being heterozygous carriers (Fig 2B and Table 5). According to ACMG/AMP guidelines, the c.1405_1406del variant is classified as LP. Her parents were unavailable for sitosterol testing.

## Discussion

In this study, we investigated the difference in sitosterol levels between normocholesterolemic and hypercholesterolemic children, and determined a serum sitosterol cutoff value to improve the overall diagnostic process of sitosterolemia. Our results show significantly increased sitosterol levels in hypercholesterolemic children, and by application of an appropriate sitosterol cutoff, 3 patients (HC1, HC2, HC3) with overtly increased sitosterol levels were identified, with molecular testing confirming sitosterolemia diagnosis in 2 patients (HC1, HC2).

Moreover, the results of our study contradict previously reported low prevalence rates of <1/1,000,000~5,000,000 [8, 9], as shown by a very high percentage (6.4%) of hypercholesterolemic children with increased sitosterol levels, in addition to 2 hypercholesterolemic patients (0.9%) being diagnosed with sitosterolemia via genetic confirmation, with one further patient also highly suspected of the disease but unavailable for molecular testing. A recent study by Brinton et al. also showed comparable results in patients with increased low-density lipoprotein cholesterol (LDL-C) concentrations (≥4.92 mmol/L [= 190 mg/dL]), with 4% of patients having sitosterol levels above the 99^th^ percentile and 0.3% having sitosterol levels consistent with sitosterolemia [14]. Although the population size and composition are quite different, both studies demonstrate the need for improvement in the diagnosis of sitosterolemia, especially considering that sitosterolemia is associated with an increased risk of cardiovascular disease [15, 16].

In physiological conditions, due to the lower affinity of NPC1L1 and SOAT2 for sitosterol compared to cholesterol, the biological absorption is very low (<5%) compared to cholesterol (~50%) [5, 17]. In sitosterolemia, pathogenic variants in the *ABCG5* or *ABCG8* genes result in defective ABCG5/ABCG8 efflux transporters [4], resulting in pathologically high absorption rates of sitosterol (15–60%) [18, 19]. Despite the structural similarity to cholesterol, sitosterol cannot be endogenously synthesized, meaning that virtually all of the sitosterol in the body must be derived from dietary sources. The usefulness of non-cholesterol sterol measurements in different forms of pediatric hypercholesterolemia has previously been reported by Noto et al. using the concept of cholesterol synthesis and absorption, and showed improved diagnostic utility especially in the detection of autosomal dominant hypercholesterolemia [20]. When applied to our findings, this indicates that the higher sitosterol levels in the hypercholesterolemic group must be a result of either increased absorption or decreased excretion, but to our knowledge, other than defective ABCG5/ABCG8 efflux transporters, there is currently no known mechanism to explain such phenomena in hypercholesterolemic children.

Due to the relative scarcity of sitosterolemia studies, a clear cutoff value to distinguish between normal and abnormal sitosterol levels was required. In our study, a reference value of 35.9 μmol/L was used as the upper cutoff value for sitosterol. This is an arbitrary calculated value (median + 5SD) derived from the sitosterol concentrations of 109 normocholesterolemic children. In an ideal study population with a larger sample size, a cutoff value would be determined by using values corresponding to the 99~99.9^th^ percentiles. However, such percentile values were deemed unsuitable for our relatively small study population, therefore an alternative method of using the median + 5SD value was selected to obtain a more reliable cutoff value. Our value of 35.9 μmol/L is in concordance with sitosterol reference ranges utilized in previous studies [9, 14], and was successfully implemented to identify 15 patients (14 hypercholesterolemic and 1 normocholesterolemic) with increased sitosterol. From this hypersitosterolemic subgroup, 3 patients (HC1, HC2, and HC3) showed overtly increased sitosterol, with molecular testing confirming sitosterolemia diagnosis in 2 patients (HC1, HC2) who gave informed consent.

The first patient (HC1) was an 18-year old girl undergoing treatment for nephrotic syndrome. Due to pathologic compensation of hypoproteinemia via increased synthesis and decreased excretion of lipids from the kidneys, hypercholesterolemia is a relatively common finding in nephrotic syndrome [21, 22]. Contrary to cholesterol, sitosterol is not known to be significantly affected by renal clearance, necessitating genetic testing to elucidate the cause for her very high sitosterol concentrations (468.1 μmol/L). Massively parallel sequencing identified two different *ABCG5* variants (c.1292C>T (p.Pro431Leu) and c.1336C>T (p.Arg446*)). Whilst the c.1336C>T nonsense variant has been repeatedly reported as a verified pathogenic variant, the c.1292C>T missense variant is classified as a VUS according to ACMG/AMP guidelines. According to the gnomAD database, the c.1292C>T variant is reported to have a minor allele frequency of 0.00041%, whilst it has not been reported in the KRGDB. Additionally, it also shows conflicting results in different ISP programs (MutationTaster: Deleterious; SIFT & Polyphen2: Tolerable). In the context of the patient’s family (Fig 2A), we can confirm patient HC1 is a compound heterozygote with two *ABCG5* variants in trans. Due to her younger brother being phenotypically normal (sitosterol 25.0 μmol/L), we could confidently assert that the c.1292C>T variant would exert a pathological function.

The second patient (HC2) was a 7-year old girl on statin treatment for suspected familial hypercholesterolemia. Her family history consisted of dyslipidemia and hypertension from her paternal grandparents, whilst her mother showed moderately increased cholesterol levels (197 mg/dL [= 5.10 mmol/L]). Furthermore, the patient also showed anemia (Hb 6.8 mmol/L [= 11.0 g/dL]). There have been previous reports of hemolytic anemia concurring in sitosterolemia patients [2], while hypercholesterolemia with poor response to statin therapy is an established indication for testing for phytosterols including sitosterol [1]. Sitosterol testing by GC-MS showed overtly elevated sitosterol levels (424.5 μmol/L), and subsequent genetic testing revealed that patient HC2 was homozygous for the c.1405_1406del (p.Ser469Glnfs*12) frameshift-inducing variant in the *ABCG8* gene. The aforementioned variant is classified as a likely pathogenic variant according to ACMG/AMP guidelines, with a minor allele frequency of 0.00041% in gnomAD database but has not been reported in the KRGDB. The parents of HC2 were heterozygous carriers for the variant.

The third patient (HC3) was a 6-month old baby girl on total parenteral nutrition (TPN) while being treated for bronchopulmonary dysplasia (BPD). She also showed signs of cholestasis, but the etiology was unconfirmed as to whether it was due to her underlying disease or associated with TPN. Cholestatic liver disease has been frequently reported in infants on long-term TPN [23, 24], pointing to the need for evaluation of the patient’s nutritional status. However, the possibility of increased sitosterol intake via TPN was deemed insufficient to adequately explain both the substantially increased sitosterol levels (278.4 μmol/L) of HC3 and the fact that other BPD patients receiving similar treatment did not show increased sitosterol levels. Unfortunately, informed consent could not be obtained from the patient’s parents at the time of the study, therefore genetic confirmation of sitosterolemia was not possible in patient HC3.

The results of our study indicate an unprecedented high frequency of both increased (14/220 = 6.4%) and overtly increased (3/220 = 1.4%) sitosterol in hypercholesterolemic children, and molecular confirmation of sitosterolemia in patients HC1 and HC2 reaffirm that such findings should not be disregarded as mere coincidence. Furthermore, serum sitosterol results of the family members of patient HC1 (Fig 2) suggest that, despite the autosomal recessive nature of sitosterolemia, obligate heterozygotes of pathogenic variants may have upper-normal to mildly elevated sitosterol levels. Interestingly, none of our patients (HC1, HC2, and HC3) with overtly increased sitosterol showed signs of xanthomatosis, which is a common finding in sitosterolemia. This further emphasizes the underestimated risk of misdiagnosis with familial hypercholesterolemia, which has been reported with increasing frequency [9, 12]. Thus, the results of our study, enforced by other previous studies [1, 14, 15, 25], strongly suggest that the prevalence of sitosterolemia is much higher than previous predictions ranging from <1/1,000,000~5,000,000.

There are some limitations to our study, with the most obvious being the small sample size of both the normocholesterolemic and hypercholesterolemic group. With our institution being a tertiary referral hospital, children visiting for routine examinations or vaccinations are a very small minority of the total pediatric patients, which leads to an innate difficulty of obtaining medically normal samples. Similarly, the recruitment criteria, which excluded patients with malignancies and/or severe congenital disorders, coupled with the aforementioned systematic and social traits associated with our hospital, rendered only a small proportion of hypercholesterolemic subjects eligible for selection. This also led to the problem of being unable to match the age distribution between the two patient groups, which is one aspect that requires improvement. As the objective of our study is to identify potential misdiagnoses, we believe the careful selection of an adequate study population using a more stringent and specific method would redeem more meaningful results than a more tolerant approach which will both yield larger sample numbers and facilitate the age-matching problem.

Additionally, although 15 children with increased sitosterol were identified, molecular testing was available for only 2 of these children and their direct family members. Thus, the remaining 1 potential sitosterolemia patient and 12 possible heterozygote carriers of *ABCG5* or *ABCG8* variants could not receive molecular confirmation. This lack of availability can be attributed to the retrospective nature of our study, which made the obtainment of informed consent and arrangements for further testing difficult, especially considering that there often was a significant time difference between the study subjects’ final hospital visit and study contact point. We intend to address this limitation in a future prospective study, whereby all suspected patients and potential carriers can undergo massively parallel sequencing.

Moreover, incomplete clinical records, especially in the hypercholesterolemic group, limited the depth of evaluation in some patients. Ideally, complete lipid profiles including LDL-C, high-density lipoprotein cholesterol (HDL-C), and triglycerides, in addition to other relevant results such as bilirubin or peripheral blood smears, should all be available when assessing hypercholesterolemia. Finally, sitosterol measurement from all patients were conducted in both different times and settings. Most samples were drawn at nonuniform times of the day, with fasting states also not accounted for, which implies that diurnal variation cannot be ruled out. However, although not specific to sitosterol, the intraindividual variation of non-cholesterol sterols has been reported to be insignificant [26], which partially alleviates limitations associated with diurnal variation. In future studies, we plan to recruit study samples over a longer timeframe, which will result in larger sample sizes without compromising the quality of the selection process, as well as obtaining complete medical records of all subjects who enroll in the study.

Our demonstration that higher sitosterol levels are observed in hypercholesterolemic children, with 2 previously unsuspected patients newly diagnosed with sitosterolemia via GC-MS and massively parallel sequencing, reaffirm the significant risk of misdiagnosis of sitosterolemia with familial hypercholesterolemia, and that the prevalence of sitosterolemia is much higher than previously thought. Similar to its oft confused counterpart, familial hypercholesterolemia, sitosterolemia is also associated with an increased risk of premature atherosclerosis and sudden cardiac death [1, 14, 15], and myocardial infarctions have been reported in patients as young as 4-years old [25]. To our knowledge, this is the first study to evaluate the association between hypercholesterolemia and increased sitosterol in the Korean pediatric population, with our results of increased (6.4%) and overtly increased (1.4%) sitosterol rates in hypercholesterolemic children significantly higher than previous studies. Based on current findings, in conjunction with the potential risks associated with increased sitosterol, special lipid testing including sitosterol, especially in children with uncontrolled hypercholesterolemia, at younger ages is recommended in order to identify and rectify potential misdiagnoses. Likewise, increased clinical suspicion and ability to recommend genetic counseling to those patients with elevated sitosterol should help predict the true prevalence of sitosterolemia.

## Supporting information

S1 Dataset (PDF)
