# Supplementary material for: High prevalence of increased sitosterol levels in hypercholesterolemic children suggest underestimation of sitosterolemia incidence
Source: PLoS One. 2020 Aug 26;15(8):e0238079. doi: 10.1371/journal.pone.0238079 (PMC7449458; doi:10.1371/journal.pone.0238079)
Supplement: S1 Dataset — (PDF) [file pone.0238079.s001.pdf]

Supplemental dataset. Phytosterol and cholesterol level of each subject

Number	Sex	Age (yr)	Hypercholesterolemia	Sitosterol (µmol/L)	Campesterol (µmol/L)	Cholesterol (mmol/L)
1	Female	5	No	4.60	2.12	3.06
2	Male	8	No	4.34	3.21	3.63
3	Male	17	No	6.99	11.67	3.37
4	Female	10	No	12.09	22.57	3.06
5	Male	4	No	6.31	10.79	3.83
6	Female	5	No	7.49	13.35	3.65
7	Male	12	No	4.82	8.64	3.78
8	Female	4	No	9.46	13.17	3.39
9	Male	4	No	14.39	17.83	4.01
10	Female	18	No	8.71	8.06	5.18
11	Male	8	No	16.14	18.73	4.84
12	Male	8	No	12.25	13.69	4.04
13	Female	5	No	17.19	18.46	4.30
14	Female	9	No	4.80	8.68	4.87
15	Male	12	No	6.10	11.70	3.19
16	Male	6	No	5.39	10.02	3.44
17	Male	17	Yes	9.07	12.25	6.19
18	Female	12	Yes	10.50	13.92	6.24
19	Female	13	Yes	6.41	6.80	7.20
20	Female	10	Yes	12.63	10.16	6.50
21	Female	7	Yes	24.78	30.64	8.50
22	Male	10	Yes	7.17	17.26	7.30
23	Female	2	Yes	17.58	20.52	6.60
24	Male	6	Yes	17.50	21.72	16.76
25	Male	16	Yes	6.70	6.73	6.94
26	Female	18	Yes	468.07	388.87	8.81
27	Male	10	Yes	18.21	30.33	7.10
28	Female	9	Yes	8.87	11.50	7.54
29	Male	9	Yes	7.41	5.34	7.07
30	Male	8	Yes	13.37	15.73	6.79
31	Male	16	Yes	8.38	8.15	7.64
32	Male	18	Yes	10.42	15.19	6.99
33	Female	11	Yes	8.47	8.51	7.25
34	Male	11	Yes	7.55	8.72	6.63
35	Female	11	Yes	8.43	12.47	9.04
36	Male	14	Yes	21.76	38.75	7.51
37	Female	18	Yes	6.06	4.77	7.15
38	Male	18	Yes	6.43	7.69	7.38
39	Female	7	Yes	424.40	267.11	7.46
40	Male	11	Yes	10.75	12.77	6.76
41	Female	4	Yes	13.68	19.18	6.68
42	Male	19	Yes	7.08	11.89	6.63
43	Male	12	Yes	8.43	17.73	7.20
44	Female	17	Yes	10.11	17.87	6.58
45	Male	10	Yes	8.68	13.94	6.68
46	Male	8	Yes	15.19	18.44	6.81
47	Male	19	Yes	11.25	18.57	7.12
48	Female	9	Yes	12.05	20.48	7.59
49	Male	12	Yes	14.03	26.93	6.53
50	Male	17	Yes	11.97	19.22	6.53
51	Female	4	Yes	20.08	24.87	8.03

52	Female	4	Yes	12.33	20.05	6.53
53	Female	9	Yes	9.16	16.08	6.66
54	Male	9	Yes	19.75	30.01	8.57
55	Male	10	Yes	15.81	23.33	8.78
56	Female	6	Yes	44.92	58.94	16.37
57	Male	7	Yes	25.02	42.82	18.21
58	Male	4	Yes	18.35	26.31	10.62
59	Male	15	Yes	8.67	13.17	6.86
60	Male	1	Yes	12.74	19.46	7.10
61	Female	16	Yes	11.36	19.55	7.02
62	Female	10	Yes	22.67	26.96	7.12
63	Female	11	Yes	19.52	32.88	7.74
64	Female	9	Yes	7.44	12.48	6.53
65	Male	18	Yes	19.74	56.05	7.46
66	Female	7	Yes	19.32	27.49	9.04
67	Male	6	Yes	22.71	37.91	10.02
68	Male	3	Yes	9.56	11.22	6.92
69	Male	7	Yes	12.25	18.15	7.56
70	Male	8	Yes	11.37	18.02	7.72
71	Male	6	Yes	5.35	24.69	8.26
72	Male	7	Yes	10.31	17.38	6.89
73	Male	8	Yes	12.09	22.59	6.60
74	Male	6	Yes	14.82	21.28	7.72
75	Male	8	Yes	8.19	12.39	6.79
76	Male	11	Yes	10.96	16.21	6.50
77	Female	18	Yes	20.22	29.19	8.88
78	Female	17	Yes	17.31	22.69	9.92
79	Male	6	Yes	21.68	40.21	12.10
80	Female	4	Yes	14.96	23.29	6.58
81	Male	17	Yes	8.98	16.20	6.86
82	Male	11	Yes	13.25	24.87	7.15
83	Female	11	Yes	9.69	17.86	6.94
84	Male	10	Yes	31.08	42.16	11.40
85	Male	12	Yes	7.92	12.52	6.63
86	Female	14	Yes	10.38	19.22	8.29
87	Male	6	Yes	30.05	21.65	8.37
88	Male	15	Yes	9.44	17.15	7.33
89	Male	5	Yes	15.26	18.42	7.15
90	Male	10	Yes	14.87	25.39	8.73
91	Female	14	Yes	9.93	21.19	7.12
92	Male	7	Yes	14.01	23.15	6.73
93	Female	1	Yes	- 0.09	1.12	8.13
94	Male	3	Yes	36.97	43.81	6.63
95	Female	17	Yes	8.52	13.80	6.99
96	Male	9	Yes	10.17	14.64	6.94
97	Female	9	Yes	11.81	21.61	7.02
98	Female	16	Yes	12.58	16.78	7.10
99	Male	5	Yes	5.58	3.47	17.79
100	Male	11	Yes	74.56	76.86	11.76
101	Female	6	Yes	10.54	22.17	6.60
102	Male	1	Yes	29.80	17.82	6.97
103	Female	18	Yes	2.76	4.86	6.58
104	Female	15	Yes	9.03	15.96	7.49
105	Male	11	Yes	17.03	36.88	6.73
106	Female	16	Yes	8.28	19.48	9.38

107	Female	1	Yes	18.71	14.82	7.56
108	Female	13	Yes	5.57	8.45	6.55
109	Male	16	Yes	15.19	21.71	7.07
110	Female	14	Yes	7.21	15.12	6.92
111	Female	18	Yes	50.49	71.12	10.75
112	Male	18	Yes	6.14	11.07	6.55
113	Female	2	Yes	12.04	15.42	11.68
114	Male	12	Yes	11.76	22.49	12.46
115	Female	3	Yes	10.21	14.80	7.28
116	Male	7	Yes	14.04	22.84	13.39
117	Male	9	Yes	13.16	19.33	7.02
118	Male	5	Yes	18.03	19.70	13.91
119	Male	6	Yes	9.21	17.17	8.29
120	Male	12	Yes	9.81	15.83	9.48
121	Female	15	Yes	11.21	16.90	6.94
122	Female	1	Yes	10.73	15.51	7.59
123	Female	8	Yes	26.07	51.42	12.48
124	Male	13	Yes	10.33	17.02	7.46
125	Male	1	Yes	11.66	19.63	10.00
126	Male	13	Yes	27.85	59.01	12.35
127	Male	16	Yes	14.41	29.15	7.98
128	Female	14	Yes	9.22	16.69	9.76
129	Male	3	Yes	8.38	14.80	7.25
130	Male	17	Yes	9.85	19.40	6.63
131	Female	8	Yes	30.88	48.18	12.82
132	Male	0	Yes	3.86	5.57	7.33
133	Male	4	Yes	15.04	19.67	7.33
134	Male	10	Yes	14.65	19.58	11.47
135	Male	7	Yes	12.68	16.36	8.52
136	Female	13	Yes	47.58	73.19	10.52
137	Male	2	Yes	21.95	21.14	15.82
138	Male	11	Yes	13.18	18.71	6.99
139	Male	13	Yes	16.68	21.47	6.81
140	Male	7	Yes	72.99	83.31	6.63
141	Male	8	Yes	37.13	57.56	13.16
142	Male	3	Yes	15.90	18.55	8.37
143	Male	10	Yes	13.39	22.45	9.12
144	Female	7	Yes	19.65	35.42	8.00
145	Male	4	Yes	6.54	11.89	8.13
146	Female	10	Yes	18.67	29.05	10.96
147	Female	5	Yes	9.50	12.64	6.53
148	Male	1	Yes	15.44	23.87	7.93
149	Male	16	Yes	10.31	22.58	9.56
150	Female	10	Yes	15.76	18.56	6.86
151	Female	11	Yes	11.66	17.92	6.53
152	Female	11	Yes	15.72	26.25	9.87
153	Male	10	Yes	27.18	51.64	14.87
154	Male	3	Yes	20.82	24.15	9.30
155	Female	8	Yes	11.39	17.63	6.60
156	Male	18	Yes	2.69	16.19	7.77
157	Female	17	Yes	14.98	12.59	6.84
158	Female	15	Yes	11.45	18.96	11.01
159	Male	14	Yes	10.02	13.12	10.26
160	Male	9	Yes	12.55	19.23	8.75
161	Female	14	Yes	11.35	14.21	9.53

162	Male	7	Yes	19.22	25.25	10.39
163	Male	16	Yes	13.76	20.61	7.49
164	Female	7	Yes	14.96	20.49	6.66
165	Female	10	Yes	73.49	53.32	9.40
166	Female	7	Yes	16.01	25.28	8.31
167	Male	10	Yes	12.19	17.63	7.64
168	Female	8	Yes	24.70	30.40	11.60
169	Male	14	Yes	12.31	17.01	8.26
170	Male	9	Yes	13.83	15.89	9.17
171	Female	17	Yes	12.82	16.24	6.48
172	Male	10	Yes	12.03	15.36	6.94
173	Male	15	Yes	11.37	14.02	7.10
174	Female	16	Yes	13.26	17.66	8.57
175	Female	0	Yes	278.30	81.73	8.21
176	Male	15	Yes	20.61	32.32	9.19
177	Male	9	Yes	23.93	34.28	18.88
178	Male	17	Yes	20.65	25.67	6.81
179	Male	11	Yes	20.99	39.96	7.67
180	Male	16	Yes	63.34	94.25	24.76
181	Male	18	Yes	11.49	15.36	7.93
182	Female	18	Yes	13.30	16.76	7.93
183	Male	6	Yes	26.08	28.17	7.28
184	Male	9	Yes	14.77	20.60	7.07
185	Male	5	Yes	18.48	28.01	9.22
186	Male	17	Yes	11.81	15.47	6.63
187	Male	4	Yes	26.17	28.70	12.28
188	Male	3	Yes	19.19	18.33	7.33
189	Female	11	Yes	11.20	13.07	6.84
190	Female	15	Yes	17.20	28.26	7.38
191	Male	5	Yes	31.91	48.87	15.82
192	Male	10	Yes	16.86	25.44	7.07
193	Female	6	Yes	18.39	21.63	9.40
194	Female	7	Yes	26.97	44.37	10.98
195	Male	1	Yes	10.07	11.72	6.50
196	Male	5	Yes	14.22	17.07	6.58
197	Male	4	Yes	15.27	18.35	7.77
198	Female	7	Yes	25.18	31.75	8.11
199	Male	17	Yes	12.73	16.67	7.36
200	Female	14	Yes	21.66	25.74	11.63
201	Female	13	Yes	46.87	27.75	7.46
202	Male	9	Yes	11.22	21.78	7.30
203	Male	4	Yes	20.09	20.42	7.30
204	Male	6	Yes	17.26	23.12	10.00
205	Male	11	Yes	11.69	9.47	6.58
206	Female	14	Yes	15.13	17.87	6.71
207	Female	11	Yes	13.99	17.50	7.61
208	Female	15	Yes	13.89	16.23	7.56
209	Female	13	Yes	14.57	16.16	7.72
210	Female	13	Yes	12.68	14.89	7.30
211	Female	6	Yes	28.15	28.73	13.47
212	Female	6	Yes	19.14	26.98	6.60
213	Female	6	Yes	16.73	17.80	6.48
214	Female	6	Yes	18.01	21.88	8.24
215	Male	10	Yes	9.68	11.75	6.79
216	Male	6	Yes	22.08	27.06	9.30

217	Male	4	No	11.63	15.48	4.07
218	Female	5	No	8.72	14.23	4.27
219	Female	3	No	11.63	16.47	5.05
220	Female	2	No	9.93	13.73	3.86
221	Female	12	No	6.54	10.73	4.27
222	Female	10	No	7.03	3.74	4.66
223	Female	4	No	15.26	17.72	4.58
224	Male	6	No	8.24	13.48	3.76
225	Female	9	No	8.48	12.73	4.64
226	Male	4	No	8.48	13.98	3.81
227	Male	9	No	7.75	12.73	4.95
228	Female	3	No	11.39	17.97	3.99
229	Female	4	No	9.45	12.23	4.25
230	Male	1	No	5.57	3.99	3.29
231	Female	7	No	11.63	16.72	4.61
232	Male	4	No	11.39	17.47	4.66
233	Female	18	No	6.54	9.48	4.35
234	Male	7	No	6.54	9.98	3.24
235	Female	7	No	8.97	12.73	4.22
236	Male	1	No	4.85	4.99	3.63
237	Female	6	No	10.90	17.47	4.64
238	Female	15	Yes	15.51	19.97	6.55
239	Female	4	Yes	12.12	16.47	6.89
240	Female	6	No	10.18	5.74	3.24
241	Female	7	No	17.45	18.72	4.43
242	Male	4	No	11.15	12.48	4.38
243	Male	1	No	11.87	10.98	4.30
244	Male	7	No	8.97	8.49	3.39
245	Male	6	No	10.90	11.73	3.86
246	Male	2	No	8.48	5.99	2.36
247	Female	3	No	12.60	11.73	3.96
248	Male	6	No	13.33	21.47	4.87
249	Female	9	No	14.78	16.97	4.69
250	Female	1	No	13.08	11.23	5.00
251	Male	4	No	12.36	7.99	4.30
252	Male	8	No	9.69	5.74	3.32
253	Male	12	No	10.42	11.98	3.89
254	Male	3	No	25.93	32.45	3.65
255	Male	3	No	13.57	12.48	3.94
256	Female	3	No	12.60	12.23	4.87
257	Male	9	No	6.78	7.49	2.77
258	Male	7	Yes	14.30	17.47	8.31
259	Female	18	Yes	27.14	40.93	11.24
260	Female	5	Yes	26.41	23.46	7.43
261	Male	13	Yes	18.17	24.21	6.73
262	Female	16	Yes	25.93	38.19	6.86
263	Male	3	Yes	32.23	37.19	7.67
264	Male	0	Yes	22.05	8.74	6.92
265	Male	6	Yes	18.41	4.49	7.69
266	Female	12	Yes	22.05	23.21	10.46
267	Female	10	Yes	19.38	21.47	7.12
268	Male	6	Yes	18.90	22.46	6.84
269	Male	6	Yes	17.45	22.46	7.46
270	Male	6	Yes	12.60	14.98	6.68
271	Female	4	Yes	18.17	17.72	6.53

272	Male	4	No	15.02	15.97	3.78
273	Male	9	No	9.69	7.49	4.66
274	Female	5	No	18.90	22.46	3.81
275	Female	5	No	16.48	15.23	3.99
276	Male	4	No	14.05	19.22	4.12
277	Male	8	No	12.36	13.48	4.12
278	Female	15	No	12.84	16.22	4.87
279	Female	8	No	12.36	14.98	3.52
280	Female	8	No	13.81	14.23	3.60
281	Male	3	No	12.60	12.48	3.03
282	Male	1	No	14.30	16.22	4.07
283	Male	16	No	11.63	16.22	3.68
284	Female	2	No	14.54	19.72	4.77
285	Male	18	No	16.96	21.47	4.33
286	Male	17	No	9.21	9.73	3.16
287	Male	14	No	9.45	18.22	3.94
288	Male	11	No	9.21	10.48	4.35
289	Male	10	No	9.69	6.74	3.65
290	Male	9	No	10.90	11.73	5.02
291	Female	8	No	14.54	20.22	3.86
292	Male	8	No	10.66	11.23	3.73
293	Female	7	No	16.96	19.97	4.84
294	Male	7	No	15.26	17.72	3.68
295	Female	5	No	24.23	34.94	4.64
296	Female	5	No	11.39	14.98	3.96
297	Female	3	No	15.26	16.97	3.68
298	Female	3	No	15.51	20.97	4.22
299	Male	3	No	12.36	13.23	4.35
300	Female	2	No	14.05	16.22	3.73
301	Male	0	No	9.21	9.98	3.47
302	Male	10	No	16.96	18.97	4.64
303	Male	6	No	13.33	17.97	3.94
304	Female	7	No	9.21	12.73	4.35
305	Male	6	No	9.69	18.72	4.87
306	Male	5	No	16.48	20.72	3.73
307	Female	9	No	9.69	8.24	3.68
308	Male	5	No	12.84	17.22	3.91
309	Male	2	No	8.48	7.74	3.81
310	Male	16	No	10.18	10.23	4.33
311	Female	4	No	19.87	13.73	3.65
312	Male	2	No	11.63	12.48	3.89
313	Male	2	No	18.17	17.22	4.25
314	Male	3	No	12.84	15.97	4.07
315	Male	9	No	15.51	19.22	4.35
316	Female	14	No	15.02	17.72	3.70
317	Male	7	No	15.51	20.72	4.51
318	Female	17	No	12.60	17.47	4.43
319	Male	7	No	10.42	13.48	3.78
320	Male	17	No	26.17	38.94	3.78
321	Male	1	No	36.83	45.18	4.48
322	Male	3	No	19.87	27.21	4.07
323	Male	2	No	12.60	15.72	3.60
324	Male	6	No	11.39	14.98	2.77
325	Male	5	No	15.99	23.21	4.43
326	Female	3	Yes	16.48	15.23	6.66

327	Male	8	Yes	22.05	27.71	6.60
328	Female	11	Yes	82.87	44.43	6.89
329	Male	4	Yes	27.62	38.44	7.07
